# Performing MHz-Level Repetition Rate Tuning for Coherent Dual-Microcomb Interferometry

**DOI:** 10.3390/mi16040448

**Published:** 2025-04-10

**Authors:** Enqi Yan, Mingliang Peng, Jian Tang, Jiyuan Huang, Donglai Tian, Suyang Liu, Zhijun Meng, Xianbin Li, Lingxiao Zhu, Shuhua Yan, Guochao Wang

**Affiliations:** College of Intelligence Science and Technology, National University of Defense Technology, Changsha 410073, China; yanenqi_96@163.com (E.Y.); pengmingliang@nudt.edu.cn (M.P.); jian_t@nudt.edu.cn (J.T.); jiyuan625@foxmail.com (J.H.); 15773147607@163.com (D.T.); liusuyang11@nudt.edu.cn (S.L.); mzj727@126.com (Z.M.); lixianbin23@nudt.edu.cn (X.L.); zhulingxiao31@163.com (L.Z.); yanshuhua996@163.com (S.Y.)

**Keywords:** dual comb, coherent, tunable repetition rate, thermal control, interferogram

## Abstract

The high-repetition-rate dual-microcomb interferometry, characterized by its high precision, rapid measurement speed, and ease of integration, shows significant promise in applications such as precision spectroscopy and high-speed precision ranging. As dual-microcomb interferometry usually requires a specific difference in repetition rates, tuning the repetition rate of the microcomb is crucial for integrating dual-microcomb sources and enhancing the measurement performance, including the precision and the update rate. This work demonstrates a coherent dual-microcomb system driven by a single continuous-wave fiber laser at 1560.49 nm. The system employs a hybrid tuning method combing single-sideband (SSB) modulation for precision pump frequency control (enabling continuous repetition rate tuning across a 4.34 MHz range) with thermal control for coarse tuning. The linear dependence between the repetition rate and pump modulation frequency shows a measured coefficient of 143.58 kHz/GHz. This method enables dual microcombs with MHz-level repetition rate tuning, significantly relaxing the fabrication and pairing requirements for microresonators. The advancement is particularly valuable for dual-comb spectroscopy and ranging applications, including gas detection and satellite formation flying.

## 1. Introduction

In recent years, optical frequency combs (OFCs) have emerged as revolutionary light sources, playing pivotal roles in various fields, including laser ranging [1,2,3], photonic microwave generation [4,5], spectroscopy [6,7], and atomic clocks [8,9]. Among these applications, dual-comb interferometry (DCI) employs two optical frequency combs with slightly different repetition rates to down-convert frequency and phase information from the optical domain to the radio-frequency (RF) domain. This enables signal acquisition, detection, and processing in the RF domain, significantly reducing the bandwidth and response speed requirements of detectors. Currently, DCI has been widely applied in dual-comb spectroscopy (DCS) [10,11,12] and dual-comb ranging (DCR) [13,14,15]. The advancement of silicon photonics technology [16] has introduced the generation of soliton microcombs (SMCs) using on-chip resonators, providing new source options for DCI. These combs are distinguished by high repetition rates, low power consumption, broad spectral coverage, and ease of integration, offering significant application potential in high-precision ultrafast ranging and high-resolution spectral detection. To maintain the coherence of the dual combs, a single continuous-wave (CW) laser is used to pump two microresonators. The resulting SMCs share the optical phase of the seed laser, forming a compact, coherent dual-comb source [17,18].

The repetition rate of the SMC is primarily determined by the dimension of the microresonator during fabrication, which makes flexible control challenging. Wafer-level fabrication generally introduces a repetition frequency error in the MHz range. In dual microcomb systems, the repetition rates of the two microcombs must exhibit specific differences. Consequently, post-fabrication testing and selecting suitable microresonator chips are required to form compatible microresonator pairs. This process increases system complexity, cost, and randomness. In dual-comb measurements, the repetition rate difference of the two SMCs determines the measurement update rate, while the repetition rate itself impacts the measurement accuracy. If the repetition rates of SMCs can be tuned, the demanding chip-matching requirements can be alleviated. Moreover, for selected combinations of microresonator chips, the adjustability of the repetition rate can enhance the system’s measurement performance.

To date, numerous studies have explored methods for tuning the repetition rate of SMCs. The Raman self-frequency shift (SFS) of DKS OFCs has been theoretically analyzed by varying the pump laser frequency, and this effect induces changes in the soliton repetition rate [19,20,21]. In a single resonator, two solitons propagating in opposite directions can have their repetition rates independently controlled through pump frequency tuning [22]. Additionally, small-range continuous tuning of the microcomb repetition rate can be achieved by introducing an auxiliary laser and varying its frequency [23]. The aforementioned methods primarily achieve repetition rate tuning by adjusting the frequency of the pump or auxiliary laser, thereby changing the relative detuning between the laser and the microcavity resonance. However, in coherent dual-microcomb systems, where two microresonators are driven by the common CW laser, independent control of a single microcomb cannot be realized through direct tuning of the CW laser. Alternatively, the SMC can be actuated by directly tuning the resonance state of the microcavity itself. By monolithically integrating AlN actuators onto $Si_{3}N_{4}$ photonic circuits, voltage control has been employed to initiate, tune, and stabilize solitons [24]. This demonstrates that piezoelectric control of the microresonator can modify the repetition rate. Additionally, a thermal-response control method has been used to generate DKS in microtube resonators, where external stress tuning controls the repetition rate of the soliton over a range of 200 kHz while maintaining the pump laser frequency [25]. However, such tuning methods require the integration of piezoelectric actuators and their corresponding drivers. Another effective approach involves thermally controlling the resonance state of the microcavity to adjust SMCs, a technique with significant application potential for systems featuring fixed pump laser frequencies or dual-comb configurations [26].

Here, we performed MHz-level repetition rate tuning for coherent dual-microcomb interferometry. In this dual-microcomb source, two silicon nitride microresonators, each with a free spectral range (FSR) of approximately 25 GHz, were driven by a single continuous wave laser at a wavelength of approximately 1560.49 nm. The system employs a hybrid repetition rate tuning method combing single-sideband (SSB) modulation for continuous fine tuning with thermal control for coarse tuning. Specifically, the fine-tuning range of 4.34 MHz is achieved through precise pump frequency control enabled by SSB technology, which ensures accurate repetition rate regulation. This capability is further augmented by coarse-tuning methods, where thermal control shifts the resonant frequency by integer multiples of its free spectral range (FSR), enabling broader repetition rate adjustments. Furthermore, dual-microcomb interferogram with various repetition rate differences were demonstrated using two silicon nitride microresonator chips, offering potential for the practical implementation of dual-comb applications across various scenarios.

## 2. Method and Principle

**Figure 1:**
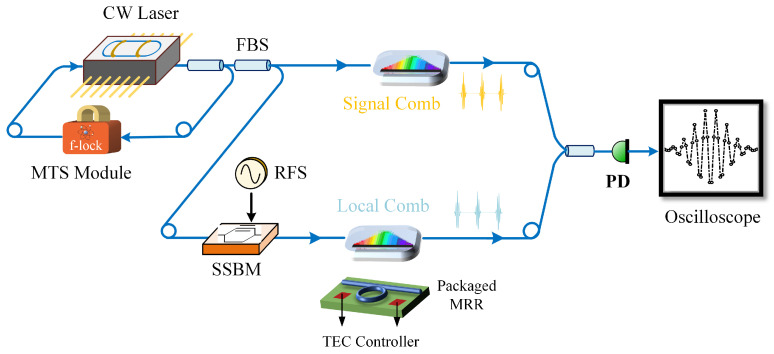
Configuration of the coherent dual-microcomb system. CW laser, continuous-wave laser; MTS module, modulation transfer spectroscopy module; RFS, radio-frequency source; SSBM, single-sideband modulator; PD, photodetector; FBS, fiber beam splitter; MRR, microring resonator.

The structure of the coherent dual-microcomb interferometry system is shown in Figure 1. A single-frequency laser, stabilized to the rubidium atomic transition line [27], is used to drive two silicon nitride microresonators, ensuring their coherence. The seed light is split into two paths: one path is directly coupled into a microresonator to generate an SMC, while the other path is first modulated by an optical modulator for radio-frequency modulation, and then coupled into another microresonator to generate a second SMC. Each silicon nitride microresonator is integrated with a packaged TEC for thermal control, which adjusts the detuning between the resonance frequency and the pump laser frequency to enable the generation of SMCs. This system structure offers several advantages: first, the two SMCs share the phase of the seed laser, ensuring their coherence; second, thermal control of the resonance frequency enables independent control of the two microcombs without altering the pump laser frequency, which would otherwise affect the generation of both comb signals simultaneously.

To generate microcombs, the pump laser frequency must reside in the red-detuned region of the microcavity resonance to achieve a single-soliton state.

According to the resonant condition of the microcavity, the longitudinal mode resonance $f_{m}$ corresponding to the mode number mm satisfies the following equation:

(1) $$f_{m}=\frac{c\cdot m}{n_{eff}L},\;m\in Z^{+}$$

where $n_{eff}$ represents the effective refractive index and *L* denotes the cavity circumference.

Thermal control is employed to adjust the relative detuning between the resonance frequency and the pump laser frequency. This thermodynamic process operates through two primary mechanisms. The first mechanism is the hermo-optic effect, which causes changes in the effective refractive index and can be characterized by the thermo-optic coefficient $\alpha_{n}$. The second mechanism is thermal expansion of the material, which modifies the geometric dimensions of the microcavity and can be characterized by the thermal expansion coefficient ε. By taking the partial derivative of Equation (1) with respect to temperature, the following result is obtained:

(2) $$n_{eff}L\frac{\partial f_{m}}{\partial T}+f_{m}L\left(\frac{\partial n_{eff}}{\partial T}+\frac{\partial n_{eff}}{\partial f_{m}}\cdot \frac{\partial f_{m}}{\partial T}\right)+f_{m}n_{eff}\frac{\partial L}{\partial T}=0$$

We can derive the relationship between the resonance and temperature variations as follows:

(3) $$\frac{df_{m}}{dT}=-\frac{f_{m}\left(\alpha_{n}+n_{eff}\varepsilon \right)}{n_{g}}$$

where $n_{g}=n_{eff}+f_{m}\frac{\partial n_{eff}}{\partial f_{m}}$ is the group refractive index.

By substituting the parameters of silicon nitride, including its effective refractive index, thermal expansion coefficient, thermo-optic coefficient, group refractive index, and pump frequency, as listed in Table 1, the theoretical value of the temperature-dependent tuning coefficient for the resonance of the silicon nitride microcavity is calculated to be −3.32 GHz/°C.

As for the repetition frequency, it can be expressed as follows:

(4) $$f_{rep}=\frac{c}{n_{g}L}$$

By taking the partial derivative of Equation (4) with respect to temperature, the following result is obtained:

(5) $$\frac{\partial f_{rep}}{\partial T}=\frac{\partial f_{rep}}{\partial n_{g}}\cdot \frac{\partial n_{g}}{\partial T}+\frac{\partial f_{rep}}{\partial L}\cdot \frac{\partial L}{\partial T}$$

We can derive the relationship between the repetition frequency and temperature variations as follows:

(6) $$\frac{df_{rep}}{dT}=-f_{rep}\left(\frac{\alpha_{n}}{n_{g}}+\varepsilon \right)$$

By substituting the parameters listed in Table 1, the theoretical value of the temperature-dependent tuning coefficient for the repetition frequency of the mircocomb is calculated to be −434.22 kHz/°C.

**Figure 2:**
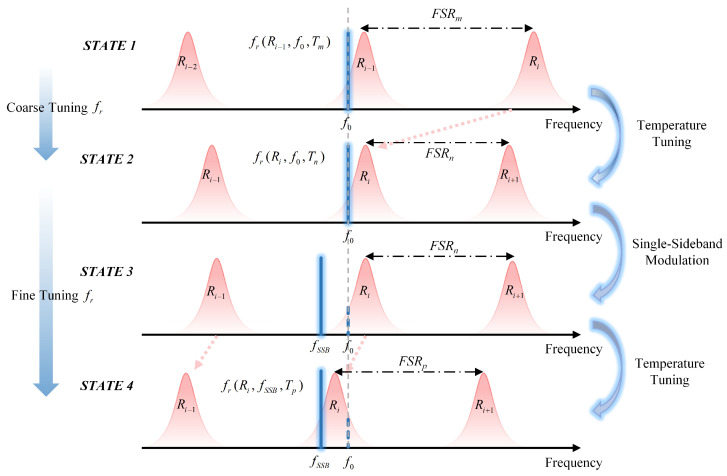
Single sideband modulation is applied to adjust the pump laser frequency, while thermal control is utilized to tune the resonance frequency of silicon nitride microresonators, facilitating the generation of SMCs.

As illustrated in Figure 2, the pump laser driving the local comb is adjusted via single-sideband modulation, while temperature tuning is utilized to control the resonance. This process enables the generation of SMC with different repetition frequencies. Specifically, in STATE 1, the pump laser with frequency $f_{0}$ drives the silicon nitride microresonator at resonance $R_{i-1}$, generating the SMC at temperature $T_{m}$ with a repetition rate of $f_{r}\left(R_{i-1},f_{0},T_{m}\right)$. The resonance frequency of the microcavity can be shifted by adjusting the temperature through the use of a TEC. With a fixed pump laser frequency $f_{0}$, the microcavity resonance $R_{i}$ is tuned into the blue-detuned region relative to the pump frequency at temperature $T_{n}$, resulting in an SMC with a repetition rate of $f_{r}\left(R_{i},f_{0},T_{n}\right)$, referred to as STATE 2 in Figure 2. When the pump frequency remains unchanged, thermal control must shift the microcavity resonance frequency by an integer multiple of the FSR to position the resonance frequency within the blue-detuned region of the pump frequency, enabling the generation of the comb. This method facilitates wide-range, discrete tuning of the optical comb repetition rate.

To achieve continuous tuning of the repetition rate while maintaining the stability of the other microcomb in the dual-comb system, the seed laser frequency is not directly altered. Instead, single-sideband modulation is applied using a continuously adjustable radio-frequency (RF) signal source to electro-optically modulate the seed laser and change the pump laser frequency $f_{SSB}$, as illustrated in STATE 3 of Figure 2. However, under this condition, the detuning relationship between the resonance and the pump laser does not satisfy the criteria for soliton comb generation. Therefore, the TEC is used to slightly adjust the resonance frequency, shifting the microcavity resonance $R_{i}$ into the blue-detuned region of the pump laser frequency $f_{SSB}$. The corresponding temperature is denoted as $T_{p}$, and the FSR of the microcavity is represented as $FSR_{p}$. The comb signal coupled out under these conditions has a repetition rate expressed as $f_{r}\left(R_{i},f_{SSB},T_{p}\right)$, referred to as STATE 4 in Figure 2.

By continuously tuning the RF frequency of the SSB modulation and thermally controlling the microcavity resonance to maintain a fixed detuning relationship, soliton comb generation is ensured, enabling repetition rate tuning across different states.

## 3. Experimental Results

### 3.1. Experimental Setup

Our experimental setup employs a single continuous-wave fiber laser as the common seed source for both microcombs, with its frequency precisely stabilized to the transition line of ^87^Rb atoms using modulation transfer spectroscopy (MTS) [27]. Precise tuning of the pump frequency for the local microcomb is achieved using a single-sideband modulator (SSBM) with 40 GHz modulation bandwidth. The half-wave voltages for the I and Q channels measure 3.7 V and 3.1 V respectively, with the RF driving power maintained at 15 dBm. The silicon nitride microresonator, with a circumference of approximately 6 mm, is thermally stabilized through integration with a TEC module capable of 0.002 °C temperature resolution, enabling both dissipative Kerr soliton generation and precise repetition-rate tuning. The signal comb and local comb combine together and are subsequently detected by a balanced photodetector. A high-speed oscilloscope operating at 1.2 GSa/s acquires the resulting beat signal for digital storage. All of the experiments are conducted under controlled environmental conditions within a cleanroom, where temperature and humidity are maintained at about 23 °C and 40%, respectively.

### 3.2. Single Sideband Modulation and Generation of SMC

**Figure 3:**
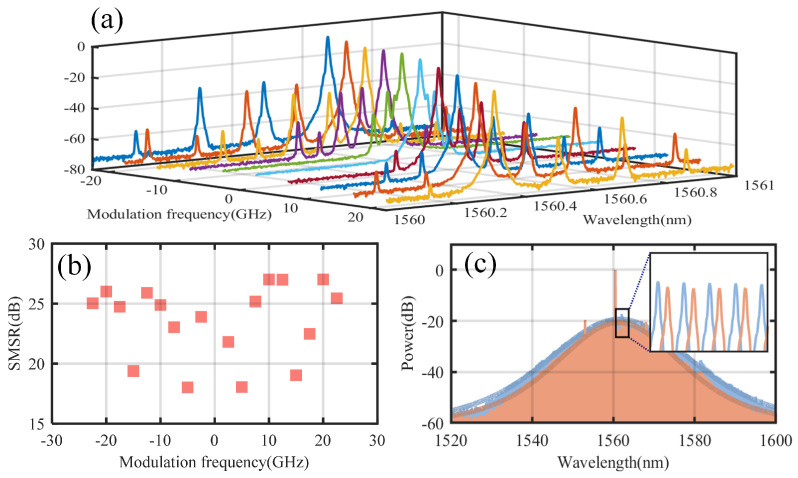
The results of single sideband modulation and the spectrum of the microcomb generated by its pumping. (**a**) The spectrum of single sideband modulation. (**b**) The side-mode suppression ratios (SMSR) at different modulation frequencies. (**c**) The optical spectra of the microcombs generated at two different single-sideband modulation frequencies.

In the proposed coherent dual-microcomb system with adjustable repetition rates, single sideband (SSB) modulation is used to adjust the pump laser frequency of the local comb, which drives the microresonator to generate SMCs. Achieving a high side-mode suppression ratio (SMSR), as shown in Figure 3b, minimizes the influence of the carrier and other sidebands within the microcavity, thereby enabling the efficient generation and stable operation of the microcomb. Figure 3c presents the optical spectra of the microcomb, generated and adjusted through thermal control, under two different single-sideband modulation frequencies.

### 3.3. Results of Repetition Rate Tuning

**Figure 4:**
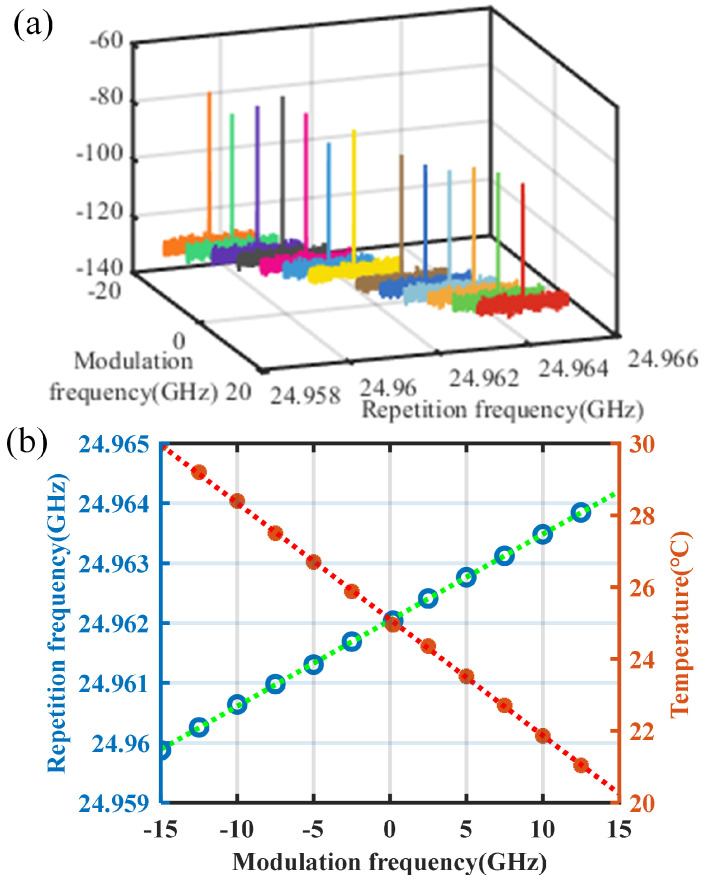
(**a**) Variations in the microcomb repetition rate corresponding to different single sideband modulation frequencies of the pump laser. (**b**) Linear fitting results illustrating the relationship between the pump laser modulation frequency, repetition rate, and temperature.

By varying the RF modulation frequency of the single sideband modulator and adjusting the temperature of the microcavity, a silicon nitride microresonator is driven to generate optical frequency combs with tunable repetition rates across MHz level, as illustrated in Figure 4. For an initial pump laser frequency $f_{0}$ = 192,121.36 GHz, the RF modulation frequency is varied within the range of [−15, 15] GHz. By controlling the TEC to adjust the resonator temperature, optical comb signals with different states are obtained, showing a repetition rate variation of 4.34 MHz. The linear fitting coefficient between the change in repetition rate and the modulation frequency shift is determined to be 143.58 kHz/GHz, aligning closely with the theoretical prediction of 130.79 kHz/GHz. Furthermore, the linear fitting coefficient between the modulation frequency and the resonator temperature in the single-soliton state is measured as −0.324 °C/GHz, consistent with the theoretical value of −0.301 °C/GHz. The observed discrepancies between theoretical and experimental results are within reasonable expectations. During the modeling process, certain parameters (e.g., pump frequency) are assigned actual system values, while others (including silicon nitride’s effective refractive index, group refractive index, thermo-optic coefficient, and thermal expansion coefficient) utilize typical literature values.

### 3.4. Dual-Microcomb Interferograms with Different Repetition Rate Differences

**Figure 5:**
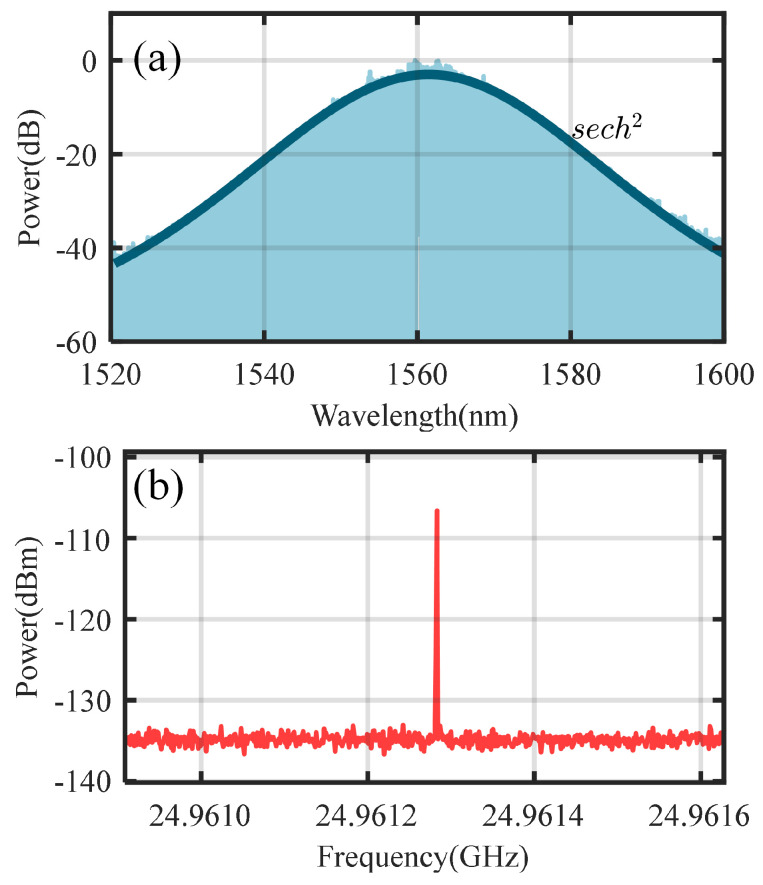
The optical spectra and the repetition frequency of the signal comb. (**a**) Optical spectra and $sech^{2}$ fit of the signal comb. (**b**) The repetition frequency of the signal comb.

**Figure 6:**
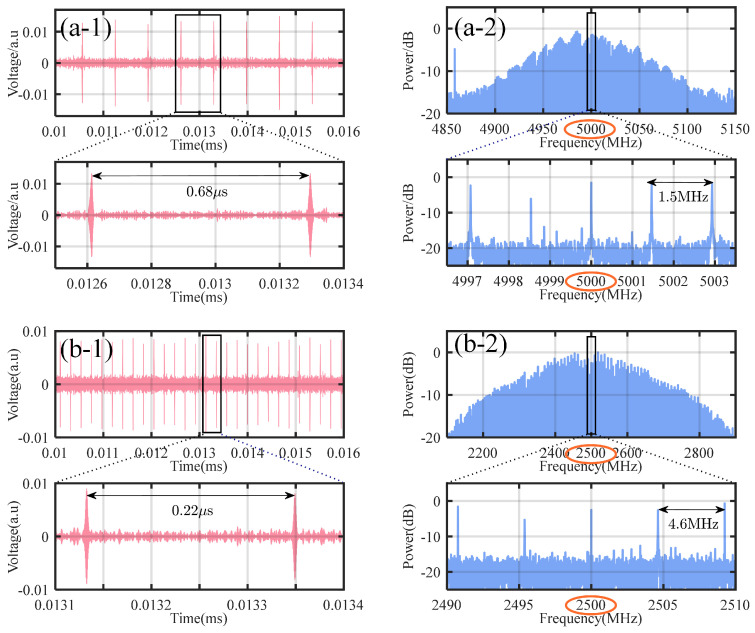
Dual-microcomb interferograms and their fourier transform results. (**a**) The local comb in $f_{r}^{LC}(R_{i},$ 5 GHz, 23.52), exhibiting a repetition rate difference of 1.5 MHz relative to the signal comb. (**b**) The local comb in $f_{r}^{LC}(R_{i-1}$, 2.5 GHz, 15.97), exhibiting a repetition rate difference of 4.6 MHz relative to the signal comb. (**a-1**) The temporal interferogram corresponding to a repetition frequency difference of 1.5 MHz. (**a-2**) The electrical spectrum obtained by Fourier transform of the temporal interferogram in (**a-1**). (**b-1**) The temporal interferogram corresponding to a repetition frequency difference of 4.6 MHz. (**b-2**) The electrical spectrum obtained by Fourier transform of the temporal interferogram in (**b-1**).

Using the configuration illustrated in Figure 1, two silicon nitride microresonators are driven to form a coherent dual-microcomb system. In this setup, the repetition rate of the local comb is adjustable, while the signal comb remains fixed for comparative demonstration, as shown in Figure 5. The repetition rate of the signal comb is represented as $f_{r}^{SC}=24.9613$ GHz. By adjusting the RF drive of the SSB modulator, the pump laser frequency of the local comb is varied. Additionally, fine adjustments using a TEC enable the generation of local combs with varying repetition rates. As shown in Figure 6, the signal comb interferes with local combs having different repetition rates. The time-domain waveform of the interferogram is recorded using a high-speed oscilloscope, and Fourier transform is applied to obtain the corresponding interference signal spectrum. From these time-domain waveforms and spectral plots, the repetition rate difference between the two combs and the RF modulation frequency of the modulator are determined. As presented in Figure 6a, the repetition rate of the local comb is 24.9627 GHz, and its single-soliton state is represented as $f_{r}^{LC}(R_{i},$ 5 GHz, 23.52). In Figure 6b, the repetition rate of the local comb is 24.9659 GHz, and its single-soliton state is denoted as $f_{r}^{LC}(R_{i-1},$ 2.5 GHz, 15.97).

## 4. Discussion and Conclusions

In summary, this study presents a hybrid tuning method enabling MHz-level repetition rate control for microcombs in silicon nitride microresonators. The method combines single-sideband modulation for precise pump frequency adjustment (enabling continuous fine repetition rate tuning across a 4.34 MHz range) with thermal control for coarse repetition rate tuning. We demonstrate a coherent dual-microcomb system powered by a single continuous-wave laser that drives two microresonators with tunable repetition rate differences. The proposed approach substantially relaxes the stringent requirements of the fabrication and pairing for microresonators in coherent dual-microcomb systems. The demonstrated technique shows significant potential for advancing dual-comb applications in spectroscopic interferometry and precision ranging.

## Figures and Tables

**Table 1:** Calculation parameters of silicon nitride microcavity [28,29].

Parameter	Symbol	Value
Effective Refractive Index	$n_{eff}$	1.99
Group Refractive Index	$n_{g}$	2.06
Thermal Expansion Coefficient	ε	$3\times 10^{-6}$/°C
Thermo-Optic Coefficient	$\alpha_{n}$	$2.96\times 10^{-5}$/°C
Circumference	*L*	6 mm
Resonance	$f_{m}$	192.1 THz
Free Spectral Range	FSR	25 GHz

## Data Availability

The data presented in this study are available upon request from the corresponding author.
